# *FHIT* Doubts are Clear Now

**DOI:** 10.1100/tsw.2010.110

**Published:** 2010-06-16

**Authors:** Anjilna Wali

**Affiliations:** Cell and Molecular Biology Laboratory, Department of Biotechnology, University of Pune, Pune, Maharashtra, India

**Keywords:** fragile histidine triad gene (FHIT), cancer, fragile regions, diadenosine triphosphate (Ap3A), apoptosis, CpG island methylation, aberrant transcripts, loss of heterozygosity (LOH)

## Abstract

The fragile histidine triad (*FHIT*) gene is a bonafide tumor-suppressor gene present on the short arm of chromosome 3 and its loss of function has been evaluated in different types of cancers. Loss of heterozygosity at various sections of the *FHIT* gene and the methylation analysis of the promoter region showed that it is one of the important and preliminary genetic alterations in the cell, and its restoration in the cell line or nude mice suppresses tumorigenicity. Current research on the *FHIT* gene has depicted that Fhit interacts with different proteins through different pathways in the nucleus, mitochondria, and cytoplasm, directing the cell to apoptosis.

Cancers arise as a result of somatic mutations, a concept dramatically reinforced by the demonstration that cellular “proto-oncogenes”, when mutationally deregulated or abnormally overexpressed, contribute to tumor formation. These genes encode proteins that govern processes of cell proliferation, differentiation, and development, but any mutations affecting their functions constitutively deregulate signaling pathways and that is why cancer cells misbehave. The discovery of genetically dominant, “activated oncogenes” also fuelled the idea that a distinct class of “antioncogenes” might oppose their effects and block tumor development. Experiments involving somatic cell fusion and chromosome segregation pointed to the existence of genes that could suppress tumorigenicity. Over the past 2 decades, many such tumor-suppressor genes have been identified, and the interplay between the oncogenes and the tumor-suppressor genes are being increasingly recognized as playing an important role in influencing the cellular kinetics of cancer. The development of human cancer is a multistep process involving the clonal evolution of abnormal cell populations that gain a selective advantage over normal cells by accumulating specific alterations in various genes; such as *p53* and *k-ras* mutations, inactivation of the *Rb* gene, and modification in *p16*, *cdc* 25, cyclin *D1*, and *FHIT*. Several nonrandom chromosomal abnormalities have been identified in chromosomes 3p, 9p, and 17p[1].

## CHROMOSOME 3 AND THE FRAGILE REGION; FRA3B

Putative oncogenes and tumor-suppressor genes are present on the short arm of chromosome 3, which includes *c-RAF-1* proto-oncogenes[2], β-retinoic acid receptor gene *(β-RAR)*, zinc finger–containing genes[3], the protein-tyrosine phosphatase-gamma gene *(PTPRG)*[4], Von Hippel Lindau *(VHP)*[5], and fragile histidine triad (*FHIT*) gene localized to 3p14.2[6] (Fig. 1). Chromosomal fragile sites are specific loci that are especially susceptible to forming gaps, breaks, and rearrangements in metaphase chromosomes when cells are cultured under conditions that inhibit DNA replication. Fragile sites are grouped into two classes: the "rare" and the "common", based on their frequency of occurrence and means of induction. The common fragile sites (CFSs) are apparently present as a constant feature in all individuals and their clinical significance is that they might predispose chromosomes to breakage and rearrangement during cancer development. The most frequently observed CFSs occur, in decreasing order, at 3p14.2 (FRA3B), 16q23 (FRA16D), 6q26 (FRA6E), 7q32 (FRA7H), and Xp22 (FRAXB). FRA3B has been of particular interest since it is the most active CFS and is located in a chromosomal band that is frequently deleted in several solid tumors, suggesting that a putative tumor-suppressor gene resides there. Based on coincident chromosomal positions of some fragile sites and cancer-specific chromosome alterations, it was suggested that fragile sites could be involved in the generation of cancer-specific chromosome breaks. Although normally stable in most somatic cells *in vivo*, numerous studies have shown that CFSs are sites of frequent chromosome breakage and rearrangements in cancer cells. The CFS-specific rearrangements most frequently observed are one or more submicroscopic deletions of several hundred kilobases directly within the CFS region, often resulting in inactivation of the associated genes[7,8]. Many studies have focused on FRA3B and FRA16D locus because they are the two most frequently expressed and well-characterized CFSs, and both lie within the large tumor-suppressor genes, *FHIT* and *WWOX*, respectively[6,9]. FRA3B is the most fragile site in the genome that extends over more than 500 kb within the ~2 Mb *FHIT* gene[6,10,11]. Consistent with its proposed function as a tumor suppressor, homozygous genomic deletions within the *FHIT* gene have also been observed in a large number of human cancers and cancer cell lines.

## STRUCTURE OF *FHIT* GENE

The short arm of chromosome 3 (3p) undergoes frequent genetic abnormalities in human malignant disease. Although four major regions of 3p (3p12, 3p21.3, 3p14.2, and 3p25) are connected to allelic loss in kidney, lung, or breast tumors, the region 3p14.2 attracts major attention due to its unique features that makes it crucial in cancer development. This region contains (1) the most fragile site in the human genome (the *FRA3B* locus), (2) the papilloma virus integration site, and (3) a familial-kidney-cancer-associated breakpoint t(3;8)(p14.2;q24), all located within one of the largest human genes, i.e., *FHIT*[6,7].

*FHIT* present on the short arm of chromosome 3 is ~2 Mb in size. It has 10 small exons that make up the 1.1-kb cDNA[12]. Exons 3, 4, and 5 are present in close proximity to the familial-kidney-tumor-associated t(3;8) translocation break, the cluster of fragile sites identified by aphidicolin-induced chromosome breaks in human-hamster hybrid cells[13]. This also includes the HPV16 integration site identified in cervical carcinoma[14] and the fragile sites in intron 5 in aphidicolin-treated hybrid cells[15]. Out of 10 exons, the first four exons and the last exon are noncoding exons; therefore, exon 5 is the first coding exon of the *FHIT* gene that extends to exon 9 and encodes a small protein of 16.8-kD mass.

After its initial discovery, there was substantial controversy on whether *FHIT* is a bona fide tumor-suppressor gene involved in the carcinogenesis process or whether its frequent alterations are merely a bystander effect due to its location at a well-known genetically fragile site, *FRA3B*. Over the years, strong evidence has developed to support the postulation that *FHIT* is a tumor-suppressor gene. The experiments that involved the transfection of *FHIT* cDNA proved that it reduced the tumorigenicity in nude mice and in the cancer cell lines as well[16,17]. Besides, studies have shown that the Fhit protein exerts its oncosuppressor activity through induction of an apoptotic mechanism that seems to be Fas-associated death domain (FADD) dependent, caspase-8 mediated, and independent from mitochondrial amplification[18].

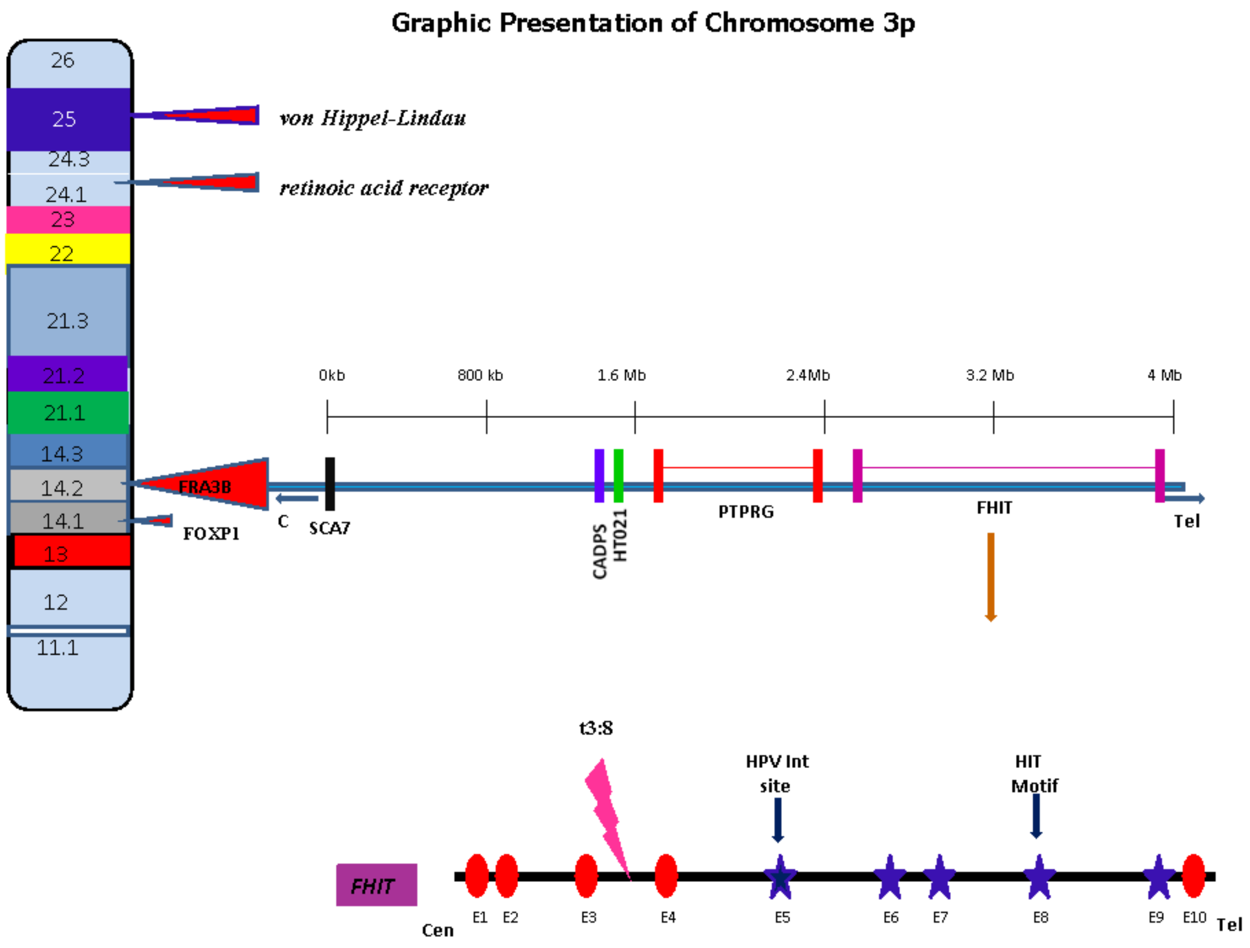

**FIGURE 1**. Chromosome 3p contains multiple numbers of potential tumor-suppressor genes, such as VHL, RAR, FOXP1, *FHIT*, etc. The short arm of chromosome 3 also contains a fragile site known as FRA3B at the 14.2 locus, which holds five genes depicted by solid-colored lines [Sca7 (black), CADPS (blue), HT021 (green), PTPRG (red), and *FHIT* gene (pink)]. Directions of centromere and telomere are shown with arrows. The *FHIT* gene is composed of 10 exons, out of which five are coding exons. Exons E1, E2, E3, E4, and E10, depicted by ⬮, are untranslated exons and exons E5, E6, E7, E8, and E9, depicted by ★, are translated exons. The t(3;8) translocation is situated between exons 3 and 4. The HPV integration site is present in exon 5 and the histidine triad residue is located in exon 8.

## Fhit PROTEIN

It was Barnes et al. who revealed that the Fhit protein encoded by the putative tumor-suppressor gene *FHIT* is a typical dinucleoside 5′,5′′′-P1,P3-triphosphate ($Ap_3A$) hydrolase that bears high structural homology with $Ap_4A$ (diadenosine tetraphosphate) hydrolase from *Schizosaccharomyces pombe*. It is believed that the loss of $AP_3A$ hydrolase activity and resulting elevated levels of $AP_3A$ or similar compounds may contribute to carcinogenesis[19]. The Fhit protein comprises 147 amino acids and has a mass of 16.8 kD. Crystallographic studies have shown that the human Fhit protein forms single orthorhombic prisms, single hexagonal needles, and pentagonal columns[20]. Within a year after the *FHIT* gene was discovered, Siprashvili et al. (1997) first reported that restoration of *FHIT* in cancer cells suppresses tumorigenicity in *in vitro* and *in vivo* xenograft experiments in nude mice[16]. In 2000, a new trend emerged in Fhit research linking the overall intracellular concentration of diadenosine polyphosphates with Fhit proapoptotic activity; thereby, the model of the Fhit-substrate signaling molecule was formed. Since then, many studies have confirmed the tumor-suppressor function of exogenous *FHIT* expression in cell lines of different origin and genetic background as well as in mouse models[21,22,23].

## *FHIT* AND APOPTOSIS

Apoptosis is a tightly regulated, physiological program characterized by specific biochemical and morphological changes executed by caspases that cleave specific substrates following activation by an apoptotic stimulus. Two major apoptotic pathways have so far been delineated: the cytoplasmic or death receptor pathway (headed by caspase-8), and the mitochondrial pathway (headed by caspase-9). Alterations in either the cytoplasmic ("extrinsic") or mitochondrial ("intrinsic") apoptotic pathways have been found in different forms of human cancers, indicating a common mechanism for tumor development and a possible hurdle for therapies aiming to exploit these systems to kill cancer cells. Studies have confirmed that *FHIT* overexpression leads to the activation of the extrinsic pathway with the activation of caspase-8, caspase-3, and PARP[24,25], as well as the intrinsic pathway by the loss of mitochondrial membrane potential[26]. In 2004, Askari and Dinh reported that the overexpression of the *FHIT* gene in cells altered the mitochondrial Delta Psi($\pi$) and enhanced the efflux of cytochrome c from mitochondria, resulting in apoptosis[27]. Thus, the observed activation pattern of both pathways induced by *FHIT* expression is in agreement with growing evidence of the presence of cross-talk between the extrinsic and the intrinsic pathways. Cisplatin-resistant cells overexpressing mitochondrial mediators of the apoptotic response, such as Bcl-2 or Bcl-xL, remained highly susceptible to cell death triggered by *FHIT* gene transfer, which specifies that the apoptotic pathway triggered by *FHIT* is independent of Bcl-2 overexpression[18]. Cell cycle distribution analysis indicated cell cycle arrest at the G(0)/G(1) phase in *FHIT*-transfected clones, indicating a retardation of cell cycle progression and increased level of Bcl-2 family members, such as Bad, Bak, and Bcl-xS[28].

Fhit has also been located in the mitochondria. This compartmentalization of Fhit could reveal a transcription-independent regulation of cell fate. Indeed, the mitochondrion is at the crossroad of numerous apoptotic pathways that synergize in triggering the morphological transitions underlying the release of proapoptotic factors into the cytoplasm. Recently, Trapasso et al.[29] have shown that Fhit interacts with various mitochondrial proteins, such as Hsp60, Hsp10, and ferridoxin reductase (FR), and the Hsp60/10 complex might be responsible for the correct folding and the import of Fhit to the mitochondria prior to the activation of the apoptotic pathway. Along with the localization of Fhit in mitochondria, the discovery of the Fhit-FR complex was also discovered. This raised the question whether reactive oxygen species (ROS) production could be associated with Fhit-mediated apoptosis. Overexpression of Fhit or induction of cellular stress in Fhit-expressing cancer cells leads to the protection of FR from proteosomal degradation, which directs the generation of ROS, followed by apoptosis. Notably, Fhit does not affect the transcriptional level of the FR gene (FDXR), but rather affects the stability of the protein[29]. Another group hypothesized the dual action of the Fhit protein in protoporphyrin IX (PpIX, a photosensitiser used in photodynamic therapy treatment)–treated cancer cells. They indicated that in the cytoplasm, Fhit-$Ap_3A$-PpIX or Fhit-AMP-PpIX serve as proapoptotic signaling molecules that promote robust cell death together with enhanced ROS production due to Fhit-FR interactions in mitochondria[30].

## *FHIT* METHYLATION AND CANCER

Although cancer is a disease driven by genetic abnormalities, recent research suggests that epigenetic alterations of gene function are also central to the pathogenesis of these diseases. Specifically, abnormal promoter region methylation in candidate tumor-suppressor genes contributes to tightly heritable gene silencing and can, thereby, cause loss of gene function, which contributes to tumorigenesis. DNA methylation involves the addition of a methyl group to position 5 of the cytosine ring; ensuing in the reduction of gene expression. Aberrant promoter methylation has been described for several genes in various malignancies and the spectrum of genes involved suggests that specific tumors may have their own distinct pattern of methylation. Genes known to date to be affected by epigenetic silencing associated with methylation include those that suppress epithelial cancers, such as breast, colon, lung, and prostate cancers, and in lymphomas and leukemias. Various tools are being used to produce epigenetic profiles of human cancer cell lines to evaluate the effects of therapeutic agents. The list of genes that are found methylated in tumors is increasing. 5' CpG island methylation of the *FHIT* gene has been investigated by various groups and it was found that the *FHIT* gene is methylated in lung, cervical, esophageal, oral, breast, and prostate cancer, along with myelodysplastic syndrome (MDS) at higher frequency. In 2008, Lin et al. showed that the *FHIT* gene was methylated in 47.2% of MDS patients as compared to normal controls, and the survival time was significantly shorter in these patients as compared to those without *FHIT* methylation. This group has specified that *FHIT* methylation occurred more frequently in advanced-stage MDS, indicating that *FHIT* methylation might be a molecular event that plays an important role in the progression of MDS[31]. The frequency of methylation of the *FHIT* gene in granulosa cell tumors (*GCTs*) of ovarian origin was 28% and it was found to be methylated in all stages of the tumor[32]. The *FHIT* gene was also found to be methylated in 15% of the prostate patients, which correlated with poor prognosis[33], 37% of primary NSCLCs (non–small cell lung cancers), and 31% of primary breast cancers[34]. Apart from the tumor tissues, 65% of lung and 86% of breast cancer cell lines were also found to be *FHIT* methylated, and it was concluded that methylation of *FHIT* is a frequent event in NSCLCs and breast cancers, and is an important mechanism for loss of expression of this gene[34]. Methylation of *FHIT* commences during lung cancer pathogenesis and may represent a marker for risk assessment. Different molecular mechanisms interplay to inactivate *FHIT* expression and support the proposition that *FHIT* methylation in normal lung tissue could represent a prognostic marker for progressive disease[35]. Along with this, the *FHIT* gene was found to be methylated in 75% of the tissue samples and 67.8% of plasma samples of hepatocellular carcinoma patients[36].

The molecular mechanisms responsible for differential susceptibility of *FHIT* hypermethylation remain unclear, but are probably related to several factors; namely, (1) difference in basal gene expression level according to cell type, (2) differential selective advantage for gene-specific aberrant methylation, (3) differential exposure to environmental carcinogens, (4) differential expression levels of transcription factors responsible for recruiting a repressor complex–containing DNA methyltransferases to a specific target, and (5) varying intrinsic susceptibility to epigenetic inactivation.

## ABERRANT *FHIT* TRANSCRIPTS IN TUMORS

*FHIT* is one of the several tumor-suppressor genes on chromosome 3, when working normally, keeping any potentially cancerous cells from growing out of control. However, when mutations or alterations eliminate one or both copies of the *FHIT* gene, the “brakes” that control cell growth are released, allowing potentially cancerous lesions to become malignant. Studies show that the *FHIT* gene loses its ability to produce its specific protein in the early stages of lung cancer[37] and the same holds true for head and neck, esophageal, colorectal, breast, and cervical cancers. The expression of *FHIT* mRNA is detectable in most normal human tissues, and the highest levels of expression are detectable in epithelial cells and tissues. The tumor-derived cell lines exhibited a pattern of products ranging from one apparently normal-sized amplified transcript to numerous aberrant bands without a normal-sized band[6]. The sequencing of the aberrant bands revealed numerous abnormal products that included the absence of various regions between exon 4 and 9, while the mRNAs of the corresponding normal tissues did not exhibit these alterations. In most of the aberrant transcripts, the beginning and the end of the deleted portions of the transcripts coincided with splice sites, suggesting that the deletions resulted from the loss of genomic regions containing or surrounding the relevant *FHIT* exons. The *FHIT* aberrant transcripts can be classified into two groups: Class I and Class II. Class I transcripts lack exon 5, which has the initial methionine codon of the *FHIT* open reading frame (ORF), resulting in the loss of the intact ORF. Class II transcripts have an intact initial methionine codon, but do not include exon 8. Thus, Class II transcripts, i.e., the wild-type ORF of the exon 8, the histidine triad-containing domain, is not present. Moreover, some of the Class II transcripts exhibited loss only of exon 8, suggesting that exon 8 was the target of deletion. Since exon 8 encodes the histidine triad motif, it is likely that neither Class I nor Class II

transcripts, constituting the major fraction of aberrant transcripts, can encode a fully functional protein. There was a report that showed that the nonfunctional *FHIT* transcripts were often produced in histopathologically normal lung tissues, which supports the theory of field cancerization, postulating that apparently normal lung tissues with primary lung cancer might have accumulated changes at the gene level[38].

## *FHIT* GENE AND LOSS OF HETEROZYGOSITY

Whether and how human tumors are genetically unstable has been debated for decades. There is now evidence that most cancers may indeed be genetically unstable and the instability exists at two distinct levels. One is at the nucleotide level, resulting in base substitutions, deletions, and insertions. The other is at the chromosomal level, resulting in loss and gain of whole or large portions of chromosomes. Recognition and comparison of these instabilities are leading to new insights into tumor pathogenesis. At the molecular level, chromosomal instability is characterized by allelic imbalance losses or gains at defined chromosomal regions. Microsatellite analysis has been widely used to detect genetic alterations in cancer. These are the most frequent genetic alterations found in virtually all kinds of tumors, if sufficient regions are examined. Microsatellite alterations may provide important prognostic information in human cancers. Loss of heterozygosity (LOH) at 3p loci, indicating chromosomal deletion, occurs very early in lung cancer pathogenesis. In 2000, Zhou et al. hypothesized that profiles of microsatellite alterations in early-stage lung cancer can be correlated with the biological behavior of the tumors and can be used as a biomarkers to predict patient prognosis[39].

LOH at the *FHIT* gene was found to be 84% in gastric cancer[40], 61% in ductal lavage of high-risk breast cancer patients[41], and 61.9% in lung cancer patients who were resected for early-stage tumor[35]. Further, it is intriguing to find that the pattern of allele loss in tumors and corresponding preneoplastic foci is identical in 89% of cases, a phenomenon referred to as allele-specific mutation[42]. It is presumed that LOH alone cannot completely suppress *FHIT* expression because many genes can be expressed monoallelicly, but at least two hits are required to inactivate tumor-suppressor genes, therefore, hypermethylation can be considered one of the hits and LOH the other.

## DIFFERENT PATHWAYS: COMMON GOAL

### *FHIT* and Catenins

Catenins are proteins found in complexes with cadherin molecules of animal cells. β-Catenin binds the cytoplasmic domain of cadherins, and loss of E-cadherin function has been implicated in cancer progression and metastasis. E-cadherin down-regulation decreases the strength of cellular adhesion within a tissue, resulting in an increase in cellular motility that in turn may allow cancer cells to cross the basement membrane and invade surrounding tissues. β-Catenin is involved in diverse cellular processes affecting morphogenesis, proliferation, differentiation, and apoptosis. Therefore, β-catenin levels and functions have to be tightly controlled within a cell. In 2007, Weiske et al investigated the role of the *FHIT* gene in the modulation of β-catenin–mediated gene transcription and they showed that Fhit directly interacts with the C-terminus of β-catenin and negatively regulates transcription of target genes[43].

### *FHIT* and Ras/Rho GTPase Regulation

Recently, Jayachandran et al.[44] demonstrated that multiple key molecules in the Ras/Rho GTPase molecular switch were negatively regulated by *FHIT* activity. The differential expression of these Ras/Rho genes and gene products modulated by ectopic activation of *FHIT* was validated by novel molecular techniques. Their finding of Ras/Rho GTPases as direct cellular targets of *FHIT* may provide a biological support for an earlier crystallographic study that suggested the *FHIT*-substrate complex to be an active signaling molecule for its tumor-suppression activity and to be functionally parallel to the Ras-GTP complexes.

## *FHIT* and the Cytoskelton

The tubulin-microtubule system plays an important role in cell division and cell proliferation. The interaction between wild-type *FHIT* or mutant *FHIT* (H96N) and tubulin *in vitro* was investigated and it was found that both wild-type and mutated forms of *FHIT* bind to tubulin strongly and specifically with $K_d$ values of 1.4 and 2.1 μm, respectively. Neither wild-type nor mutant *FHIT* cause nucleation or formation of microtubules, but in the presence of microtubule-associated proteins, both wild-type and mutant *FHIT* promote assembly to a greater extent than do microtubule-associated proteins alone, and the microtubules formed appear normal by electron microscopy. Therefore, it was suggested that *FHIT* may exert its tumor-suppressor activity by interacting with microtubules, and it was also indicated that the interaction between *FHIT* and tubulin is not related to the $Ap_3A$ hydrolase activity of *FHIT*[45]. On the other hand, the assembly of Rho GTPases has been shown to act as a molecular switch to control an actin cytoskeleton–associated oncogenic signaling transduction pathway in cancer cells through their interaction with multiple target or effector proteins. Our findings suggest that the *FHIT*-targeted down-regulation of the Ras/Rho GTPases may be mediated through a negative regulation of actin cytoskeleton–associated signaling transduction by a direct or indirect *FHIT*-Rho GTPase protein interaction[44]. *FHIT* has been shown as a target of phosphorylation by Src protein kinase; Src protein phosphorylates Y114 of *FHIT in vitro* and *in vivo* and therefore provides important clues to biochemical mechanisms involved in *FHIT* signaling[46].

## *FHIT* and Akt-Survivin Pathway

Akt is involved in cell survival pathways by inhibiting apoptotic processes. Akt is also able to induce protein synthesis pathways and is, therefore, a key signaling protein in the cellular pathways that lead to general tissue growth. Akt has been implicated as a major factor in many types of cancer. *FHIT* has been shown to modulate the Akt-survivin pathway by inhibiting the activity of Akt, a key effector in the phosphatidylinositol 3-OH kinase (PI3K) pathway. Loss of endogenous *FHIT* expression caused increased Akt activity *in vitro* and *in vivo*, which leads to the inhibition of apoptosis. The results of the study indicated that the Fhit Y114 residue plays a critical role in Fhit-induced apoptosis, occurring through inactivation of the PI3K-Akt-survivin signal pathway[47]. Further studies need to be done in order to get the clear picture of the actual signaling pathway going on in the cell.

## *FHIT* Interacts with Ubc9

Ubc9 is the conjugating enzyme of the SUMO enzymatic cascade that catalyses various post-translational modifications, such as nuclear-cytosolic transport, transcriptional regulation, apoptosis, protein stability, response to stress, and progression through the cell cycle. In 2000, Shi et al. were the first group to identify and confirm that Fhit interacts with Ubc-9 *in vivo*, and they hypothesized that since yeast Ubc9 is involved in the degradation of S- and M-phase cyclins, Fhit may be involved in cell cycle control through its interaction with human Ubc9[48]. Later in 2004, it was demonstrated that hUbc9 interacts with Fhit in an $Ap_3A$-dependent manner and suppresses the enzymatic activity of *FHIT*. The active signaling form of *FHIT* is the Fhit-substrate complex; it may be assumed that hUbc9-induced inhibition of Fhit activity should lead to elongation of the signaling complex lifetime. On the other hand, formation of hUbc9-Fhit-

$Ap_3A$ complexes may constitute the Fhit antitumor signaling pathway. The Ubc9 protein, may also mediate the nuclear localization of Ubc9-Fhit-$Ap_3A$ complex and once the Fhit is in the nucleus, it can directly affect the expression level of genes, including caspase genes[49].

## CONCLUSION

Finally, basic knowledge at the molecular level has extremely important clinical implications with regard to early diagnosis, risk assessment, and prevention by therapeutic agents. To make an impact on the curability of cancers, we need to understand the basic biology of the disease, and apply this knowledge to the development of novel diagnostic and therapeutic approaches. The molecular abnormalities may serve as the starting points for innovative treatments designed to restore the altered functions to normality. The regions of genomic instability associated with FRA3B are much larger than previously believed. This large region of defined genomic instability offers an explanation for why 3p14.2 has been shown to be involved in a variety of cancers. Loss of *FHIT* transcripts, high methylation status, and loss of one or both the alleles of the *FHIT* gene fuels the development and progression of various types of cancers. Moreover, data on apoptosis triggered by the Fhit protein showed involvement of both cytoplasmic and mitochondrial pathways. Fhit activates caspase-8 and caspase-2, which causes the release of cytochrome c and finally induces apoptosis. Fhit also sensitizes the low-affinity $Ca^{(2+)}$ transporters of mitochondria and potentiates the effect of apoptotic agents. Future studies for the identification of the molecular machinery may provide ways to act on apoptotic cell death and its derangement in cancer. These molecular alterations have the potential to be exploited for early detection and risk assessment of cancer. It is believed that LOH alone cannot completely suppress *FHIT* expression because many genes can be expressed monoallelicly, and two hits are required to inactivate tumor-suppressor genes and hypermethylation should be considered as one of the hits. Moreover, different molecular mechanisms interplay to inactivate *FHIT* expression and support the proposition that *FHIT* loss and methylation could represent a prognostic marker for progressive disease.

Understanding the interaction of Fhit with several other cytoplasmic and mitochondrial proteins, such as catenins, Ubc9, caspases, and Ras/Rho GTPases, can open up new episodes in understanding cancer progression that would allow us to identify novel cancer therapeutic targets and develop pathway-targeted molecular cancer therapy agents. Fhit may be considered as the first line of defense against the earliest stages in cancer development, and Fhit replacement or therapeutic activation of the Fhit pathway could contribute to cancer prevention. Correlation of *FHIT* tumor-suppressing function to the regulation of apoptosis, tumor cell proliferation, progression, and metastasis has provided new insight into the molecular mechanism of *FHIT* action, which opens up an era of hope.
